# Burnout syndrome among healthcare professionals in the Fako division, Cameroon: Impact of physical activity and sleep quality

**DOI:** 10.3934/publichealth.2023054

**Published:** 2023-10-16

**Authors:** Elysée Claude Bika Lele, Jerson Mekoulou Ndongo, Ako Vera Ashu-akoh, Ahmadou, Jessica Guyot, Pierre Tchienrg Moueleu Ngalagou, Bienvenu Bongue, Nicholas Tendongfor, Clarisse Noel Ayina Ayina, Marie Yvonne Lobe Tanga, Samuel Honoré Mandengue, Peguy Brice Assomo Ndemba

**Affiliations:** 1 Department of Animal Biology, Faculty of Science, University of Douala, Cameroon. PO Box 24157 Douala, Cameroun; 2 Physiology and Medicine of Physical Activities and Sports Unit, University of Douala, Cameroon. PO Box 7064 Douala, Cameroun.; 3 Department of Public Health and Hygiene, Faculty of Health Sciences, University of Buea, Cameroon; 4 Laboratoire SAINBIOSE INSERM U1059, Université JEAN MONNET, Saint-Étienne, France; 5 Faculty of Medicine and Biomedical Sciences, University of Yaounde 1 Yaounde, Cameroon

**Keywords:** burnout syndrome, healthcare professionals, physical activity, sleep

## Abstract

**Objectives:**

Burnout syndrome (BOS) is an affection mostly resulting from chronic job-related stress. Many studies have identified job-related and non-job-related factors associated with BOS. Our aim of this study was to assess the level of BOS in private and public hospital healthcare providers in Fako division, Cameroon and evaluate the impact of physical activity and sleep quality (SQ).

**Methods:**

The study was carried out in five randomly selected hospitals in Fako Division over a three-month period. Consenting doctors, nurses and laboratory technicians were recruited using consecutive sampling methods. Sociodemographic and professional characteristics were collected using a structured questionnaire. BOS was assessed using the Maslach Burnout Inventory Human Services Survey (MBI-HSS). Sleep quality (SQ) and physical activity (PA) were assessed using the Pittsburgh Sleep Quality Index (PSQI) and Global Physical Activity Questionnaire (GPAQ) respectively. Odd ratios and 95% confidence intervals were calculated and a statistical significance was set for *p*-value < 0.05.

**Results:**

The mean age was 32 ± 6 years and 70.9% female. BOS prevalence was 66.3% with 71.4% in females and 53.9% in males (*p* = 0.002). Of the 232 participants with BOS, 65.7%, 52% and 53.7% had moderate to high emotional exhaustion, depersonalization and decreased personal accomplishment, respectively. Moderate to high PA as well as poor SQ were not significantly associated with BOS while longer sleep duration (>8 h) was associated with a greater odd of BOS.

**Conclusions:**

The prevalence of BOS was high among healthcare professionals. While PA showed no protective effects, high sleep duration could increase its risk.

## 1. Introduction

Burnout syndrome (BOS) is a serious consequence of chronic exposure to work-related stressors among service professions such as teaching and healthcare [1],[2]. The World Health Organization (WHO) defines burnout as a syndrome resulting from chronic workplace stress that has not been successfully managed [3]. It is characterized by three dimensions: Feelings of energy depletion or exhaustion, increased mental distance from one's job or feelings of negativism related to one's job and reduced professional efficacy [3]. How chronic stress leads to BOS was postulated by the expanded model of BOS which suggested that BOS results when an individual is unable to adequately handle repeated stressors [4],[5]. Factors identified to be associated with BOS include: Work overload, lack of social support, insufficient rewards and professions such as teaching and healthcare [6]. Burnout has been associated with negative outcomes. Some of these adverse outcomes include reduced employee organizational commitment, lower productivity and performance, reduced engagement, employee ill health, increased absenteeism and depression and increased intention to abandon one's job. Those who are burned out can also negatively affect their colleagues through increased personal conflict and disruption of the tasks assigned to them [7],[8].

BOS is of greater concern among healthcare professionals. Population-based studies show a wide variation of it prevalence between 13% to 80% with highest prevalence in healthcare professionals in developing countries and lowest in the general population [9],[10]. Among healthcare professions, the prevalence ranges between 7% to 80%, with the highest prevalence reported among medical doctors [11]. In sub Saharan Africa with healthcare professional to patient ratio of less than 1/1000, BOS is more prevalent among nurses with a prevalence of up to 80% [1],[12]. A study in Malawi suggested that up to 2/3^rd^ of healthcare workers suffer from burnout mostly due to emotional exhaustion [13].

Prolonged BOS among employees causes several physical, psychological and occupational consequences on their job. Reduction of work output and efficacy, poor sleeping and decreased cognitive functions and abilities are some of the consequences of BOS among healthcare professionals [14]. Moreover, higher workload and shifted work faced by many healthcare workers can depress their sleep duration and quality and lead to BOS. There is a dearth of data on the impact of quality of sleep and physical activity on burnout. In Cameroon, with physician/patient ratio of 1:12500 [12] coupled with the increase socio-political instability and the Anglophone crisis in the Northwest and Southwest regions, there is a paucity of data on the prevalence, associated factors of burnout syndrome and the impact of quality of sleep and physical activity on BOS. Our aim of this study was to assess the frequency of BOS among healthcare professionals in Fako division Cameroon and determine his association with physical activity and sleep quality.

## 2. Methods

### 2.1. Study design and population

This was a cross-sectional hospital-based comparative study in Fako division Southwest region Cameroon. Healthcare professionals were approached and self-administered questionnaires were used to collect data on experience of BOS and factors associated with it as well as their sleep quality and practice of physical activity.

The Fako division is one of the six divisions in the South West Region of Cameroon with Limbe being its administrative capital. According to 2005 Cameroon national institute of statistics report, the Fako division has a surface area of 2093 km^2^ and a total population of 466412 [15]. We conducted our study in 3 public and 2 private randomly selected hospitals. The doctors, nurses and laboratory technicians in the selected hospitals were then recruited using consecutive sampling proportionate to size. The minimum sample size calculated was 334 using a prevalence of 68% found among teaching staff of university institutions in Douala [16] and 5% precision. Ethical clearance was obtained from the Faculty of Health Sciences Institutional Review Board and informed consent was obtained from the participants prior to the enrolment. Participants were met individually in their office and invited to participate to the study. Participants with at least six months of continuous activity were included in the study.

### 2.2. Data collection

Data was collected from March to June 2020, using a structured questionnaire made up of four sections. The first section contained information on socio-demographic characteristics, work and personal characteristics. The second section was assessed BOS, the third section assessed sleep quality and the fourth section assessed level of physical activity

### 2.3. Parameters measurements

BOS was assessed using the Maslach Burnout Inventory for human services survey (MBI-HSS). This is a reliable, widely used and validated tool for assessing burnout among healthcare personnel worldwide [17]. The MBI-HSS is made up of 22 items (numbered from 1 to 22) divided into three subscales; emotional exhaustion (EE) with 9 items, depersonalization (DP) with 5 items and personal accomplishment (PA) with 8 items. The answers given by the respondents were based on a 7-likert scale: Never (0), sometimes a year or less (1), once a month or less (2) a few times a month (3) once a week (4) several times a week (5), every day (6). Each subscale was classified as low, moderate and high. BOS was classified as low, moderate and high and level of BOS was high when there was a higher mean score of EE and DP and a lower mean score of PA [17]. Because MBI-HSS had been used previously in other African countries [1],[13],[18] such as Nigeria, Ethiopia, South Africa and even in Cameroon, we found it appropriate for our study.

Quality of sleep was assessed using the Pittsburgh sleep quality index (PSQI). The PSQI is a self-rated questionnaire which assesses sleep quality, duration and disturbances over a one-month time interval. It is made up of nineteen individual items. These nineteen items measure sleep quality along seven components, including subjective sleep quality, sleep latency, sleep duration, habitual sleep efficiency, sleep disturbances, use of sleeping medication and daytime dysfunction. In scoring the PSQI each of the seven components is scored zero (no difficulty) to three (severe difficulty). The sum of the scores for these seven components yields one global sleep score (range 0 to 21). A Pittsburgh sleep quality index score of greater than five is considered to be indicative of poor sleep quality [19].

Level of physical activity was assessed using the global physical activity questionnaire (GPAQ). This questionnaire was developed by the World Health Organization (WHO) for physical activity surveillance in 2002 as part of the WHO STEPwise Approach to Chronic Disease Risk Factor Surveillance (STEPS) [20]. It collects information on physical activity participation in three settings or domains (including physical activity at work, travel to and from places and recreational activity) as well as sedentary lifestyle. It is made up of sixteen questions (P1 to P16). Participants were classified as low, moderate and high physical activity level.

### 2.4. Statistical analysis

Data were collected and analyzed using SPSS version 20. Quantitative variables were presented as means ± standard deviation and qualitative variables were presented as frequencies and percentages. Quantitative data were compared between gender and type of hospital using the student t-test while qualitative data were compared using the chi-squared test. Binary logistic regression was used to determine factors associated with BOS. Odd ratios and 95% confidence intervals were calculated and statistical significance was set at p-value less than 0.05.

## 3. Results

### 3.1. Study Overview

Of the 378 participants that were met, 21 (6%) refused to participate to the study and 7 (2%) questionnaires were excluded because of incomplete data. Three hundred and fifty (350) participants were included in the study. The mean age of participants was 32 ± 6 years, 70.9% were female and 50.3% were single. Table 1 shows demographic, professional, physical activity and sleep characteristics of the participants. Nurses were the most represented with 60.3% (*n* = 211) and the majority of participants were working in the service of Medicine (40%). Of the 350 participants, 51.4% (*n* = 180) were from private hospitals and 48.6% (*n* = 170) were from public hospitals. Around 58% (*n* = 203) had worked for at most 5 years. Around 40% of participants had low level of PA moderate and high level of PA represented 29.4% and 30.6%, respectively. The mean PSQI score was 6.3 ± 1.5 and 52% of participants had a PSQI score equal or higher than six indicative of poor sleep quality. The mean sleep duration was 6.5 ± 3.2 hours and short (<6 h) and long (>8 h) sleep duration represented 28.3% and 26.6% respectively.

**Table 1.** Characteristics of study participants.

**Project**		**Frequency**	**%**
**Age (years)**	<25	26	7.4
	25–34	197	56.3
	35–44	117	33.4
	≥45	10	2.9
**gender**	Male	102	29.1
	Female	248	70.9
**Marital Status**	Single	176	50.3
	Married	174	49.7
**Job category**	Nurse	211	60.3
	Doctor	76	21.7
	Lab worker	63	18.0
**Number of children**	0	156	44.6
	1	44	12.6
	≥2	150	42.9
**Health facilities**	Private	180	51.4
	Public	170	48.6
**job seniority (years)**	<5	203	58.0
	5–10	123	35.1
	>10	24	6.9
**Working unit**	Emergency	33	9.4
	Medicine	140	40.0
	Paediatry	27	7.7
	Surgery/Maternity	99	28.3
	Laboratory	51	14.6
**Physical activity**	Low	140	40
	Moderate	103	29.4
	High	107	30.6
**PSQI score**	<6	168	48.0
	≥6	182	52.0
**Sleep duration (h)**	<6	99	28.3
	6–8	158	45.1
	>8	93	26.6

Note: PSQI: Pittsburg sleep quality index.

### 3.2. Prevalence of BOS and its components

**Figure 1.**
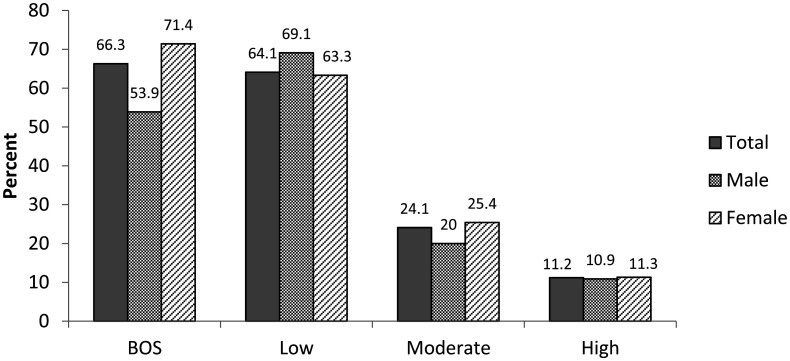
Prevalence and level of BOS according to gender.

**Figure 2.**
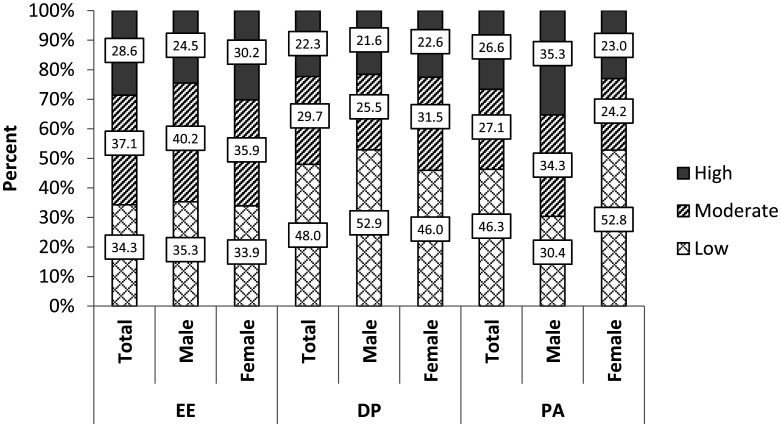
Components of BOS according to gender.

Figures 1 and 2 show prevalence, severity and components of BOS in the sample. Data are compared between male and female. Of the 350 participants, 232 (66.3%) had BOS (Figure 1). Of this, 64.7% (*n* = 150) had low BOS, 24.1 % (*n* = 56) had moderate BOS and 11.2% (*n* = 26) had high BOS. BOS was significantly higher in female compared to male (*p* < 0.05) while no significant difference was observed for BOS severity (*p* < 0.05). Of the 232(66.3%) participants with BOS, 28.6%, 37.1% and 34.3% reported high, moderate and low emotional exhaustion respectively. Also 22.3%, 29.7% and 48% reported high, moderate and low depersonalization, respectively, while 26.6%, 27.1% and 46.3% reported high, moderate and low personal accomplishment (Figure 2). Low PA was significantly frequent in female (52.8%) compared to male (30.4%), *p* = 0.001 while no significant association was found between EE and DP with gender (*p* = 0.539 and *p* = 0.443, respectively).

### 3.3. Factor associated with BOS

Tables 2 and 3 show association of BOS with sociodemographic and professional factors as well as with physical activity and sleep characteristics. The risk of BOS was significantly greater in female gender [*OR*: 2.13, *95%CI* (1.32–3.43), *p* = 0.002], married participants [*OR*: 1.7, *95%CI* (1.09–2.67), *p* = 0.02], participants with two or more children [*OR*: 1.79, *95%CI* (1.11–2.89), *p* = 0.017], public hospitals [*OR*: 1.98, *95%CI* (1.25–3.13), *p* = 0.013] and income disssatisfaction [*OR*: 2.85, *95%CI* (1.65–4.91), *p* < 0.0001] (Table 2). BOS was significantly associated with moderate PA compared to low PA [*OR*: 2.6, *95%CI* (1.46–4.65), *p* = 0.001] but no significant association was found of intense PA. Also, when combining moderate with intense PA compared with low PA, the difference was not significant. BOS was not significantly associated with poor sleep quality while it was significantly associated in participants with long sleep duration (>8 h) compared short sleep duration (<6 h) [*OR*: 2.58, *95%CI* (1.37–4.87), *p* = 0.003].

**Table 2.** Association between Sociodemographic and professional factors with BOS.

**Project**		**No BOS**		**BOS**		***OR (95% CI)***	***p***
**Variable**	**Modalité**	***N***	**%**	***N***	**%**		
**Age (yrs)**	<25	10	38.5	16	61.5	ref	0.719
	25–34	69	35	128	65	1.16 (0.5–2.69)	0.731
	35–44	35	29.9	82	70.1	1.46 (0.61–3.54)	0.398
	≥45	4	40	6	60	0.94 (0.21–4.17)	0.932
**Gender**	Male	47	46.1	55	53.9	ref	
	Female	71	28.6	177	71.4	2.13 (1.32–3.43)	0.002
**marital**	Single	69	39.7	105	60.3	ref	
	Married	49	27.8	127	72.2	1.7 (1.09–2.67)	0.02
**profession**	Doctor	24	31.6	52	68.4	ref	0.705
	Nurse	69	32.7	142	67.3	1.22 (0.45–3.3)	0.692
	Lab	25	39.7	38	60.3	1.03 (0.52–2.04)	0.935
**Number children**	0	64	41.0	92	59.0	ref	0.036
	1	12	27.3	32	72.7	1.86 (0.89–3.87)	0.100
	≥ 2	42	28.0	108	72.0	1.79 (1.11–2.89)	0.017
**health facility**	Private	74	41.1	106	58.9	ref	
	public	44	26.1	126	73.9	1.98 (1.25–3.13)	0.003
**job seniority (years)**	<5	75	36.9	128	63.1	ref	0.022
	5–10	31	25.2	92	74.8	1.74 (1.06–2.86)	0.029
	>10	12	50	12	50	0.59 (0.25–1.37)	0.217
**income satisfaction**	Yes	36	53.7	31	46.3	ref	
	No	82	29	201	71	2.85 (1.65–4.91)	<0.0001
**Working unit**	Emergency	14	42.4	19	57.6	ref	0.322
	Medicine	43	30.7	97	69.3	0.74 (0.3–1.82)	0.512
	Paediatry	13	48.1	14	51.9	1.23 (0.63–2.42)	0.548
	Surgery/Maternity	30	30.3	69	69.7	0.59 (0.23–1.52)	0.272
	Laboratory	18	35.3	33	64.7	1.25 (0.61–2.57)	0.535

Note: BOS: burnout syndrome; OR: odd ratio; CI: confidence interval

**Table 3.** Association of physical activity and sleep quality with BOS.

**Project**		**No BOS**		**BOS**		***OR (95%CI)***	***p***
		***N***	**%**	***N***	**%**		
**Physical activity**	**Low**	58	41.4	82	58.6	ref	0.005
	**Moderate**	22	21.4	81	78.6	2.6 (1.46–4.65)	0.001
	**Intense**	38	35.5	69	64.5	1.28 (0.76–2.16)	0.345
**PSQI score**	**<6**	49	29.2	119	70.8	ref	
	**≥6**	69	37.9	113	62.1	0.68 (0.43–1.05)	0.084
**Sleep duration (h)**	**<6**	41	41.4	58	58.6	ref	0.011
	**6–8**	57	36.1	101	63.9	1.25 (0.75–2.1)	0.392
	**>8**	20	21.5	73	78.5	2.58 (1.37–4.87)	0.003

## 4. Discussion

In this study, we determine the prevalence and level of BOS among healthcare professionals in the Fako division, to identify factors associated and determine the impact of quality of sleep and physical activity. We reported an overall burnout prevalence of 66.3%, where 65.7% had moderate to high EE, 52% had moderate to high DP and 46,3% had low PA. BOS was higher among healthcare professionals in public hospitals and occurred more frequently among females. Factors significantly associated to BOS were female gender, married status, having more than one child, health facility, job seniority and income disssatisfaction. Contrary to what expected, BOS was not significantly associated with sleep quality and physical activity practice did not show a protective effect.

The prevalence of BOS found in our study was similar to that reported by Kim et al in Malawi, and Ojedokun et al in Nigeria. They reported a prevalence of 62% and 66.4% respectively [21],[22]. However, our prevalence was higher than 42% that reported by Mandengue et al in Douala Cameroon on general practitioners [18]. The increase in the prevalence of BOS could be as a result of the recent socio-political unrest in the southwest and northwest regions of Cameroon in which healthcare professionals have to face added stress in carrying out their duties. Moreover, the COVID-19 context in which the study was realized could also contribute to the high prevalence of BOS. Likewise, our prevalence was also higher than that reported by Bhagavathula in Ethiopia whose prevalence was 13.7% [23]. This probably was due to the fact that their study was conducted only in one health facility. With respect to severity, 11.2% of our participants had high level of burnout syndrome while 24.1% and 64.7% had moderate and low levels respectively. This was similar to that reported by Madede et al in Mozambique [24]. They reported 11.1%, 17.9% and 71% for high, moderate and low levels of burnout, respectively. Over 20% of participants with BOS reported high emotional exhaustion, depersonalization and personal accomplishment. Similar findings were reported in 2018 by Kim et al in Malawi [21].

Gender has been observed to influence the prevalence of burnout. Many studies have observed that burnout is higher among males than females [25]–[27]. We, however, reported a high prevalence of burnout among females than males. Our finding was similar to that reported by Madede et al, and Mutale et al, [24],[28]. This high prevalence among females could be as a result of the dual role performed by women as responsible workers in their place of work as well as caring mothers, wives and housekeepers at home. Other work-related factors which are more prevalent in women and may be associated with stress include: lower education, lower income and harassment. However, the percentage of high level of personal accomplishment was significantly lower in female compared to male participants. This can be due to the fact that women are less self-assured than men. Furthermore, to feel accomplished, confidence matters as well as competence.

In our study, healthcare professionals from public hospitals suffered more from burnout syndrome mostly from emotional exhaustion when compared to their counterparts in private hospitals. Similar result was reported by Coetzee et al in South Africa where they explained that healthcare professionals in private hospitals have a more organized working environment hence less prone in developing BOS [29]. Also, healthcare professionals in public hospitals have a higher workload when compared to private hospitals. Many patients go to public hospitals because it is relatively cheaper when compared to private hospitals. This results in work overload for healthcare professionals in public hospitals and consequent exhaustion.

Although a higher prevalence of burnout has been reported among nurses followed by doctors [30],[31], we did not find any significant difference among doctors, nurses and laboratory scientists/technicians. This could be due to the fact that all the health professionals were exposed to similar working conditions. Nevertheless, the prevalence of BOS found among nurses in our study was around two times higher than what was reported in a recent meta-analysis of 94 studies which found a 30% prevalence of BOS among nurses [32]. These contrary results can be explained by the fact that in Cameroon, nurses have a very important work load with around 0.67 nurses for 1000 patients [33]. Moreover, with the Anglophone crisis and the fear of arm attacks, the number of healthcare personnel has been critically reduced in the southwest region. Besides, the study was conducted during COVID-19, which has been shown to be associated with an increasing trend of BOS [34]. All these conjunction of factors can explain the prevalence of BOS found in our study.

Various studies have reported contradictory results between BOS and marital status [30],[35],[36]. We, however, found higher level of burnout among the married than the single which was statistically significant. This was similar to that reported by Lasebikan et al and Ozumba et al in Nigeria [1],[37]. This could be because marriages come with added roles and responsibilities which could be stressful and contribute to the development of BOS. Our study showed that working experience of less than five years was significantly associated with BOS. This is similar to that reported by Mandengue et al among general practitioners in Douala [18].

In our study, 48% of the participants were poor sleepers. This is similar to that reported by Olawale et al [38]. Out of the 257 participants who sleep for less than eight hours, 61.9% had burnout syndrome. However, this result was not statistically significant. Healthcare professionals face increase pressure and stress levels due to increase demand in caring for the sick. This increased demand results in changes in their sleep and work habits. It has been shown that chronic inability to recover from stressful situations by way of sleep may lead to burnout [39]. Despites, the association between sleep quality and burnout syndrome was not statistically significant. This can be due to the fact that over time healthcare professionals become accustom to their working environment and learn to improve their sleep habits. Other studies have shown significant association of poor sleep quality with BOS risk in medical students in India [39] and in Turkish nurses [40]. On the other hand, sleep duration of greater than eight hours was significantly associated with burnout. This is quite the contrary of other studies which show a dose-dependent relation between short sleep duration and BOS risk and stress [41],[42]. However, oversleeping can be a sign of an underlying problem and regularly sleeping for longer than eight hours can shorten life expectancy [43]. A direct relationship between long sleep duration and BOS has not been established in the literature and our study did not focus on medical problems or other clinical characteristics that can help to explain this result. The subjectivity of the PSQI in the determination of sleep quality could also play a role in the results found in this study. Further studies with longitudinal design and different tools to quantify the sleep quality and duration are necessary to understand their relation with BOS.

Contrary to what expected, physical activity did not show a protective effect on BOS. We found an increased risk of BOS in participants with moderate physical activity level while of higher level the difference was not significant. This contrasts with a study in the United States which proposed that physical activity had a protective effect on BOS [44]. This could be partially due to the context of our study and to the tool used to assess the level of physical activity. Actually, the GPAQ mainly assess physical activities at home, at work and during leisure. Due to the socio-political context of the region, healthcare professionals spend more time in hospital and most of their physical activity is due to their job demand. Therefore, participants with much investment in their work are more susceptible to develop BOS and are the same time the ones with higher physical activity level. Yet, a recent longitudinal study has demonstrated that off–job physical activity (recreational and domestic) is negatively associated with primary and secondary BOS symptoms [45]. Our results are in line with previous findings on physicians in Douala [18], while another study on university teachers in Douala has shown a significant association of physical activity and reduction of BOS risk [16]. Despite contradictory results, Mandengue et al, [18] described BOS as a vicious cycle that starts with physical exhaustion aggravated by environmental factors which in turn leads to mental exhaustion and where any attempt to physically exert the body will lead to more exhaustion, hence BOS.

## 5. Strengths and limitations

To the best of our knowledge, this is the first study in Cameroon to assess the impact of quality of sleep on burnout. Our study included all major healthcare professionals compared to other studies that were limited to just nurses or doctors.

Due to the cross-sectional design of our study, we cannot draw conclusions on cause on effect of sleep quality and physical activity on BOS. Despite, how study brings in light the BOS burden in the rude context of war and COVID-19 in the south-west region of Cameroon and will contribute of the orientation of further studies to help curve the course of BOS.

## 6. Conclusion

There was an important prevalence of BOS among healthcare professionals in the Fako division in Cameroon, particularly in females. Several factors are associated with BOS although no significant association was found with sleep quality and physical activity level did not show a protective effect.

## Use of AI tools declaration

The authors declare they have not used Artificial Intelligence (AI) tools in the creation of this article.
